# Vitamin B_12_ and folate status in Spanish lacto-ovo vegetarians and vegans

**DOI:** 10.1017/jns.2019.2

**Published:** 2019-02-26

**Authors:** Angélica Gallego-Narbón, Belén Zapatera, Laura Barrios, M. Pilar Vaquero

**Affiliations:** 1 Department of Metabolism and Nutrition, Institute of Food Science, Technology and Nutrition (ICTAN-CSIC), José Antonio Novais 10, 28040 Madrid, Spain; 2 Statistics Department, Computing Center (SGAI-CSIC), Pinar 19, 28006 Madrid, Spain

**Keywords:** Vitamin B_12_, Folate, Vegetarians, Vegans, Supplementation, Hcy, homocysteine, HHcy, hyperhomocysteinaemia, MCH, mean corpuscular Hb, MCV, mean corpuscular erythrocyte volume, MMA, methylmalonic acid

## Abstract

Studies on the nutritional status of vegetarians in Spain are lacking. Prevention of vitamin B_12_ deficiency is the main concern, as dietary sources are of animal origin. The present study aimed to evaluate vitamin B_12_ and folate status of Spanish vegetarians using classical markers and functional markers. Participants were adult and healthy lacto-ovo vegetarians (forty-nine subjects) and vegans (fifty-four subjects) who underwent blood analyses and completed a FFQ. Serum vitamin B_12_, homocysteine (Hcy), methylmalonic acid (MMA), erythrocyte folate and haematological parameters were determined. The effects of the type of plant-based diet, and the intake of supplements and foods were studied by a FFQ. Mean erythrocyte folate was 1704 (sd 609) nmol/l. Clinical or subclinical vitamin B_12_ deficiency was detected in 11 % of the subjects (MMA>271 nmol/l) and 33 % of the participants showed hyperhomocysteinaemia (Hcy>15 *µ*mol/l). Regarding plant-based diet type, significantly higher Hcy was observed in lacto-ovo vegetarians compared with vegans (*P* = 0·019). Moreover, use of vitamin B_12_ supplements involved an improvement of vitamin B_12_ status but further increase in erythrocyte folate (*P* = 0·024). Consumption of yoghurts was weakly associated with serum vitamin B_12_ adequacy (*P* = 0·049) and that of eggs with lower Hcy (*P* = 0·030). In conclusion, Spanish vegetarians present high folate status but vitamin B_12_ subclinical deficiency was demonstrated using functional markers. The lack of influence of dietary sources on functional markers and the strong effect of vitamin B_12_ supplement intake emphasise the need of cobalamin supplementation in both lacto-ovo vegetarians and vegans.

Plant-based diets have been reported to provide several health benefits, reducing the risk of atherosclerosis, CHD, type 2 diabetes and the metabolic syndrome^(^^1^^,^^2^^)^. However, these diets may also increase the risks of nutritional deficiencies^(^^3^^–^^5^^)^.

Vitamin B_12_, or cobalamin, acts as a cofactor of the enzymes methionine synthase, acting in the conversion of homocysteine (Hcy) to methionine, and methylmalonyl-CoA mutase, that produces succinyl-CoA from methylmalonyl-CoA, the active form of methylmalonic acid (MMA)^(^^6^^)^. These reactions are involved in the methionine cycle and the folate cycle, both essential for DNA and RNA synthesis, erythropoiesis and the production of neurotransmitters^(^^1^^,^^7^^,^^8^^)^. Therefore, deficiencies of vitamin B_12_ and folate can result in neurological damage, due to an inhibition of the formation of the myelin sheath, and megaloblastic anaemia^(^^1^^)^.

Clinical deficiency of vitamin B_12_ can cause megaloblastic anaemia, which is characterised by high mean corpuscular erythrocyte volume (MCV) and erythrocyte distribution width, and low erythrocyte count^(^^9^^,^^10^^)^. Nevertheless, an early detection of vitamin B_12_ deficiency is a challenge, as there is not a single standard marker but several markers should be analysed for diagnosis^(^^11^^)^. In this regard, although serum vitamin B_12_ has been widely used to assess cobalamin deficiency, it can remain normal under functional deficiency conditions and false positives and negatives are common^(^^12^^,^^13^^)^. A more specific marker of functional vitamin B_12_ deficiency is Hcy, which increases under cellular vitamin B_12_ deficiency. Nevertheless, Hcy is also elevated under deficiencies of folate or vitamin B_6_, and by diets rich in methionine^(^^6^^,^^14^^–^^16^^)^. Hyperhomocysteinaemia (HHcy) promotes the formation of active oxygen species and the release of inflammatory mediators, and therefore it is considered a risk factor for CVD^(^^10^^,^^16^^–^^18^^)^. Finally, MMA represents the most specific marker for vitamin B_12_ deficiency, as it is independent of folate status and increases in cobalamin deficiency conditions before the appearance of clinical signs^(^^6^^,^^19^^)^.

Cobalamin is synthesised exclusively by bacteria and archaea, but it is accumulated along the food chain, and animal foods including meat, milk, eggs, fish and shellfish are considered the main dietary sources^(^^1^^,^^20^^)^. Other food sources are scarce and include several mushroom species like shiitake (*Lentinula edodes*), black trumpet (*Craterellus cornucopioides*) and golden chanterelle (*Cantharellus cibarius*), and certain algae and cyanobacteria such as *Chlorella* and spirulina (*Arthrospira platensis*). These are frequently used to produce tablets consumed as cobalamin supplements by vegetarians and particularly vegans^(^^21^^)^. Nevertheless, it has been observed that these supplements also contain high amounts of inactive analogues of vitamin B_12_^(^^21^^–^^23^^)^. Therefore, vitamin B_12_ deficiency is considered an extended problem for vegetarians, especially for vegans, if nutritional cobalamin supplements are not consumed^(^^4^^,^^24^^)^.

Very limited information exists on the nutritional status of vegetarians in Spain^(^^25^^)^. It is estimated that they represent at least 1·5 % of the Spanish population^(^^26^^)^. In the present study we evaluate the vitamin B_12_ and folate status of Spanish vegetarians using markers of clinical deficiency (serum vitamin B_12_ and haematological markers) and subclinical deficiency (Hcy, MMA and erythrocyte folate), constituting the first complete study on vitamin B_12_ and folate status in this population. The associations of the studied markers with the type of diet (lacto-ovo vegetarian or vegan), and the intake of vitamin B_12_-rich products and supplements were also addressed.

## Materials and methods

### Study design and participants

The study followed a cross-sectional design. Healthy lacto-ovo vegetarian or vegan adults (age ≥18 years) were recruited through advertisements in web pages in the area of Madrid, Spain. Exclusion criteria were: occasional meat or fish consumption, diagnosed digestive, renal, haematological, endocrine or oncological diseases, eating disorders, pregnancy, lactation and menopause. Subjects who had donated blood in the 3 months prior to the study were also excluded. A total of 207 subjects were initially interested, of which forty-four declined to participate and fifty-eight did not meet the inclusion criteria. In all, 105 volunteers were selected, and finally 103 completed the study and underwent all the biochemical analyses. None of the selected volunteers reported any diagnosed illness and they did not consume any medication in the days prior to blood extraction.

The present study was conducted according to the guidelines laid down in the Declaration of Helsinki and all procedures involving human subjects were approved by the Clinical Research Ethics Committee of Hospital Puerta de Hierro (Majadahonda, Spain) under the reference number 06.17 and the Ethics Committee of the Spanish National Research Council (CSIC). Written informed consent was obtained from all the participants.

### Procedure, blood sampling and biochemical assays

Volunteers attended the Human Nutrition Unit of ICTAN-CSIC between 08.00 and 08.30 hours after a 12 h fasting period. Height of the subjects was measured, and body weight, body composition and BMI were obtained using the body composition monitor Tanita BC-601 (Tanita Ltd). Blood samples were collected in Vacuette Z Serum Sep Clot Activator and K3 EDTA tubes (Greiner Bio-One GmbH). Serum was separated by centrifugation in a Jouan CR-312 centrifuge (Jouan Ltd) at 1000 ***g*** for 15 min and stored at −80°C. Vitamin B_12_ and Hcy were analysed in 100 *µ*l and 20 *µ*l of serum, respectively, by competitive immunoassay of direct chemiluminescent technology in an ADVIA Centaur XP autoanalyzer (Siemens Healthineers). Serum samples for MMA determination were prepared by solid-phase extraction using Bond Elute strong anion exchange cartridges (100 mg bed/1 ml) from Agilent. The analysis was performed using a liquid chromatography–tandem MS method previously optimised by our research group^(^^25^^)^. Erythrocyte count, erythrocyte distribution width, packed cell volume, MCV, Hb and mean corpuscular Hb (MCH) were determined in whole blood using an ADVIA 2120 flow cytometer (Siemens Healthineers). Erythrocyte folate was analysed from whole-blood samples that were refrigerated, diluted 1:25 in folate lysing agent (Beckman Coulter, Inc.), mixed, protected from light for 90 min, and finally measured by an automated chemiluminescent immunoassay in a UniCel DxI 800 Immunoassay System (Beckman Coulter, Inc.). The following quality controls from BioRad Laboratories were used: Lyphocheck Immunoassay Plus Control (for vitamin B_12_ and erythrocyte folate determinations), Lyphocheck Homocysteine Control (for Hcy determination), Lyphocheck Anemia Control (for vitamin B_12_ determination) and Liquicheck Hematology Control (for erythrocyte count, erythrocyte distribution width, packed cell volume, Hb, MCV and MCH determinations). ClinChek Controls from LETI laboratories were used for MMA determination. Quality controls were within the expected range for all measurements.

### Definitions

The proportion of individuals that presented inadequate levels within each group was defined considering the following reference levels of normality: vitamin B_12_ (>150 pmol/l), Hcy (5–15 *µ*mol/l), MMA (<271 nmol/l), erythrocyte folate (>305 nmol/l), erythrocyte distribution width (<14 %), MCV (80–96 fl), MCH (>27 pg), Hb (>130 g/l for men and >120 g/l for women), erythrocyte count (>3·6 × 10^12^/l for men and >4·2 × 10^12^/l for women) and packed cell volume (>41 % for men and >36 % for women)^(^^16^^,^^27^^,^^28^^)^.

### FFQ

A FFQ previously validated and applied for vegans^(^^29^^)^ was modified in order to estimate the composition of the diet of the Spanish vegetarian subjects. The questionnaire was completed by 103 participants. Volunteers were classified as lacto-ovo vegetarians or vegans and their consumption of supplements of vitamin B_12_ and folic acid was also recorded. The subjects were asked to indicate the frequency of consumption of each food item as consumed: never or rarely, two to four times a month, two to three times a week, four to six times a week, once per d and two or more times per d. The intake of supplements was classified according to the following frequency categories: never, one to twelve times a year, two to five times a month, two to six times a week, and daily consumed. Vitamin B_12_, folic acid and multivitamin supplements were considered. Subjects were considered supplement users if they consumed supplements at least two to five times a month. Food items included in the study of vitamin B_12_ status were: animal milk, cheese, eggs, yoghurts and vegetable milks. Mushrooms and vitamin B_12_-rich algae were not considered due to their low rate of consumption (less than 10 % of the subjects reported an occasional intake). The consumption of yoghurts was assessed through three different items (natural or flavoured yoghurts, non-fat yoghurts, other yoghurts) and vegetable milks through six items (soya milk, almond milk, oat milk, coconut milk, rice milk and cashew milk).

### Statistical analysis

#### Sample size justification

Sample size was calculated for the main variable, serum vitamin B_12_. Previous reports indicate mean values of 209 (sd 47) and 172 (sd 59) pmol/l for lacto-ovo vegetarians and vegans, respectively^(^^30^^)^. Therefore, to detect as significant a difference of 15 % between the two diet option groups, an allocation ratio of 1, a two-sided *P* = 0·05 and statistical power of 0·80, a total of eighty-eight volunteers was needed. Calculations were performed using G* Power 3^(^^31^^)^. This number was increased by 15 % to ensure a sufficient sample size in case several participants decided not to complete the study. Results are presented for 103 volunteers, with forty-nine lacto-ovo vegetarians and fifty-four vegans.

#### Data analysis

Mean and standard deviation were calculated for each of the markers. Normality was studied by the Kolmogorov–Smirnov test and non-normal variables were transformed according to their distribution. Vitamin B_12_, folate, MCV and MCH were log-transformed, while MMA and erythrocyte distribution width were transformed using the inverse function. The effect of supplementation was only studied for vitamin B_12_ supplements, as only one volunteer reported the intake of folate supplements and multivitamins were consumed occasionally for only ten individuals. Differences owed to vitamin B_12_ supplementation and diet option were studied (fixed factors) by general linear models of the transformed variables including sex and age (random factors). The age effect was not significant and therefore this variable was not included in further analyses. According to cut-off levels for each parameter, binomial variables classifying each individual as adequate or inadequate were calculated. These results are presented within diet (lacto-ovo vegetarian or vegan) and supplementation (vitamin B_12_ supplement user or non-user) groups. Furthermore, we analysed the differences in the frequency of consumption of the studied food items between subjects with adequate levels and subjects with inadequate levels of serum vitamin B_12_, Hcy and MMA through contingency tables. When independent food items were analysed (animal milk, cheese, eggs), Monte Carlo two-sided tests with 10 000 simulations and a CI of 99 % were used to provide reliability to the analysis. Yoghurt items, as well as vegetable milk items, were converted in multiple response sets and considered as food groups for the analysis, which was performed using Pearson's *χ*^2^ tests. Significance was set at *P* < 0·05 and all the statistical analyses were performed in SPSS 24 (SPSS Inc.).

## Results

### Population characteristics

The participating volunteers were young adults, with a mean age of 30·3 (sd 7·7) years, and only two subjects were older than 46 years. Most of the volunteers were women, 78 % of the total (Table 1). In relation to diet option, 47·6 % of the participants were lacto-ovo vegetarians and 52·4 % were vegans. They were normoweight with slightly higher BMI in men than women. Hcy, erythrocyte count, erythrocyte distribution width, packed cell volume and Hb were significantly different between men and women (*P* < 0·001 in all cases). Vitamin B_12_ supplements were consumed by 72·8 % of the participants.

**Table 1.** Characteristics of the studied subjects (Mean values and standard deviations; numbers and percentages)

	Women (*n* 80)		Men (*n* 23)		All (*n* 103)		
	Mean	sd	Mean	sd	Mean	sd	Normality cut-off
Age (years)	29·6	7·5	32·8	8·0	30·3	7·7	
Vegans							
*n*	42		12		54		
%	52·5		52·2		52·4		
Vitamin B_12_ users (%)	71·2		78·3		72·8		
BMI (kg/m^2^)	22·3	3·2	24·0	3·9	22·6	3·4	18·5–24·9
Vitamin B_12_ (pmol/l)	344·7	199·7	280·8	74·9	330·4	181·2	>150·0
Hcy (*μ*mol/l)	12·8	3·1	14·9	8·2	13·9	5·1	5·0–15·0
MMA (nmol/l)	157·0	79·8	222·3	236·4	171·6	133·2	<271·0
Erythrocyte folate (nmol/l)	1713·5	609·8	1670·8	617·7	1704·0	608·8	>305·0
Erythrocyte count (10^12^/l)	4·4	0·3	4·9	0·4	4·6	0·4	>3·6*, >4·2^†^
Erythrocyte distribution width (%)	13·5	1·3	12·8	0·5	13·4	1·2	<14·0
Packed cell volume (%)	40·9	3·0	45·5	2·6	41·9	3·5	>41*, >36^†^
MCV (fl)	91·9	5·4	92·3	4·7	92·0	5·2	80·0–96·0
Hb (g/l)	135	11	154	9	139	13	>130*, >120^†^
MCH (pg)	30·3	2·1	31·3	1·6	30·5	2·1	>27·0

Hcy, homocysteine; MMA, methylmalonic acid; MCV, mean corpuscular erythrocyte volume; MCH, mean corpuscular Hb.

* Cut-off for men.

† Cut-off for women.

### Diet and supplementation effects

Significant differences were found between vitamin B_12_ users and non-users, with higher levels of vitamin B_12_ (*P* < 0·001) and erythrocyte folate (*P* = 0·024), and lower MMA (*P* = 0·012) and Hcy (*P* = 0·015) in volunteers consuming vitamin B_12_ supplements (Table 2). Vegans presented significantly lower erythrocyte count (*P* = 0·032) and erythrocyte distribution width (*P* = 0·003), and higher MCV (*P* = 0·036) and MCH (*P* = 0·023) compared with lacto-ovo vegetarians. A significant interaction diet option × supplementation was found for Hb (*P* = 0·034). Means of all these haematological variables were in the normal range.

**Table 2.** Levels of the studied markers according to diet option and vitamin B_12_ supplementation (Mean values and standard deviations)

	Lacto-ovo vegetarians						Vegans						P*	
	Vitamin B_12_ users (*n* 37)		Vitamin B_12_ non-users (*n* 12)		All (*n* 49)		Vitamin B_12_ users (*n* 38)		Vitamin B_12_ non-users (*n* 16)		All (*n* 54)			
	Mean	sd	Mean	sd	Mean	sd	Mean	sd	Mean	sd	Mean	sd	Diet option	Supplement use
Vitamin B_12_ (pmol/l)	313·8	108·9	258·6	91·2	299·0	106·3	325·6	91·0	230·6	70·9	296·9	95·5	NS	<0·001
Hcy (*μ*mol/l)	13·1	3·1	14·5	3·1	13·4	3·2	12·9	3·5	13·2	2·5	13·0	3·3	0·019	0·015
MMA (nmol/l)	132·4	42·3	218·9	110·3	152·7	73·3	148·4	56·1	186·1	110·8	159·6	77·3	NS	0·012
Erythrocyte folate (nmol/l)	1753·4	564·0	1311·3	436·6	1645·2	565·0	1732·4	519·3	1659·1	634·0	1710·2	551·2	NS	0·024
Erythrocyte count (10^12^/l)	4·7	0·4	4·5	0·3	4·6	0·4	4·5	0·4	4·6	0·3	4·5	0·4	0·032	NS
Erythrocyte distribution width (%)	13·5	1·2	13·5	0·7	13·5	1·1	13·2	1·0	13·0	0·4	13·1	0·9	0·003	NS
Packed cell volume (%)	42·2	3·8	41·3	2·3	42·0	3·5	41·8	3·5	42·1	3·8	41·9	3·6	NS	NS
MCV (fl)	91·0	5·5	91·5	3·9	91·1	5·1	93·6	4·5	92·0	3·6	93·1	4·3	0·036	NS
Hb (g/l)	140	15	135	8	139	14	139	14	141	11	140	13	NS	NS
MCH (pg)	30·6	1·5	29·7	1·6	30·4	1·6	31·1	1·7	30·8	1·2	31·0	1·6	0·023	NS

Hcy, homocysteine; MMA, methylmalonic acid; MCV, mean corpuscular erythrocyte volume; MCH, mean corpuscular Hb.

* Differences were analysed through general linear models. The diet option x vitamin B_12_ supplementation interaction was only significant for Hb (*P* = 0·034).

**Fig. 1.**
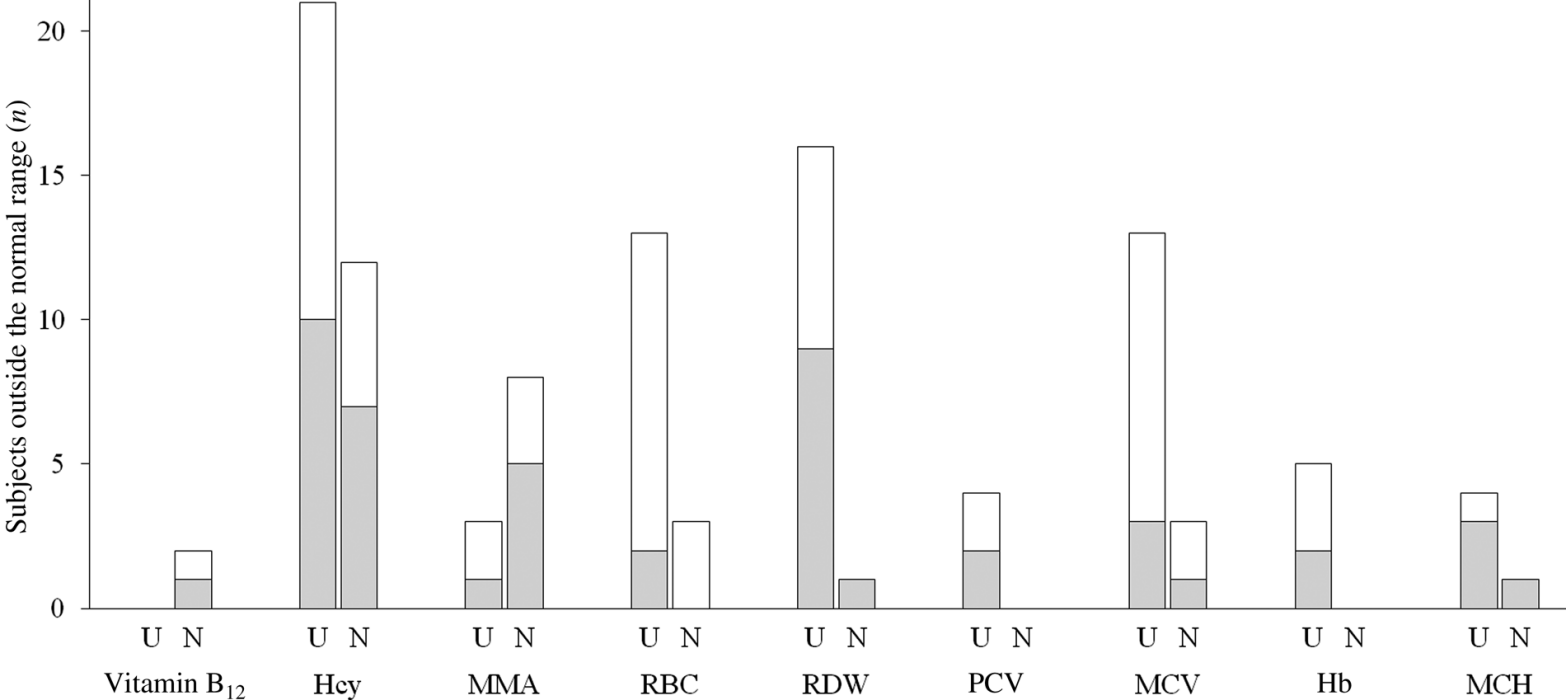
Number of subjects outside the normal range for the studied markers (according to normality cut-offs shown in Table 1). The mean corpuscular erythrocyte volume (MCV) segmented bar shows the number of individuals with values above normality. Erythrocyte folate was above the cut-off level in all the subjects and therefore it is not shown. (░), Lacto-ovo vegetarians; (□), vegans; U, vitamin B_12_ users; N, vitamin B_12_ non-users; Hcy, homocysteine; MMA, methylmalonic acid; RBC, erythrocyte count; RDW, erythrocyte distribution width; PCV, packed cell volume; MCH, mean corpuscular Hb.

Two participants presented vitamin B_12_ levels below the cut-off value of 150 pmol/l that indicates clinical deficiency (Fig. 1), while eleven subjects presented elevated MMA. HHcy was detected in thirty-three individuals. The prevalence of HHcy in vitamin B_12_ non-users (twelve subjects, 43 % of the group) was higher than in vitamin B_12_ users (twenty-one subjects, 28 % of the group). Erythrocyte count was low in sixteen subjects (15·5 % of the volunteers) and erythrocyte distribution width in seventeen subjects (16·5 % of the volunteers). Erythrocyte count low values were more frequent in vegans than lacto-ovo vegetarians (fourteen subjects, 25·9 % of vegans, *v.* two subjects, 4·1 % of lacto-ovo vegetarians). MCV was elevated in sixteen subjects, most of them vegans (twelve subjects, 22·2 % of vegans, *v.* three subjects, 6·1 % of vegetarians). Two of these subjects also presented elevated MMA and Hcy. High erythrocyte distribution width was observed mainly in vitamin B_12_ users (sixteen subjects, 21·3 % of users, *v.* one subject, 3·6 % of non-users).

The number of subjects with inadequate levels in association with the frequency of consumption of dairy products and eggs is presented in Table 3. Serum vitamin B_12_ sufficiency was associated with the consumption of yoghurts (*P* = 0·049), and Hcy was associated with egg consumption (*P* = 0·030). MMA did not present an association with any of the considered food items.

**Table 3.** Adequacy of cobalamin markers regarding food frequency consumption (Number of answers recorded for each food item or food group)

	Animal milk (*n* 103)						Vegetable milks (*n* 618)†						Yoghurts (*n* 309)‡						Cheese (*n* 103)						Eggs (*n* 103)					
	B_12_		Hcy		MMA		B_12_		Hcy		MMA		B_12_		Hcy		MMA		B_12_		Hcy		MMA		B_12_		Hcy		MMA	
Frequency of consumption	A	I	A	I	A	I	A	I	A	I	A	I	A	I	A	I	A	I	A	I	A	I	A	I	A	I	A	I	A	I
Never	86	1	59	28	77	10	334	7	227	114	307	34	253	2	173	82	226	29	60	1	44	17	56	5	62	1	45	18	58	5
2–4 times per month	8	0	7	1	8	0	124	4	90	38	112	16	17	4	16	5	18	3	15	0	10	5	13	2	16	1	7	10	13	4
2–3 times per week	2	1	2	1	2	1	58	1	41	18	54	5	7	0	3	4	6	1	16	0	9	7	14	2	15	0	13	2	14	1
4–6 times per week	2	0	1	1	2	0	30	0	21	9	26	4	10	0	8	2	10	0	3	0	3	0	3	0	5	0	4	1	5	0
Once per d	0	0	0	0	0	0	42	0	31	11	38	4	14	0	8	6	14	0	4	1	3	2	4	1	3	0	1	2	2	1
Two or more times per d	3	0	1	2	3	0	18	0	10	8	15	3	2	0	2	0	2	0	3	0	1	2	2	1	0	0	0	0	0	0
P*	NS		NS		NS		NS		NS		NS		0·049		NS		NS		NS		NS		NS		NS		0·030		NS	

B_12_, vitamin B_12_; Hcy, homocysteine; MMA, methylmalonic acid; A, adequate levels; I, inadequate levels (see the ‘Definitions’ section and Table 1).

* Differences in the frequencies of consumption between subjects with A levels and subjects with I levels of serum vitamin B_12_, Hcy and MMA (Pearson's *χ*^2^ tests).

† ‘Vegetable milks’ is the total of answers for the following food items: soya milk, almond milk, oat milk, coconut milk, rice milk and cashew milk.

‡ ‘Yoghurts’ is the total of the answers for the following food items: natural or flavoured yoghurts, non-fat yoghurts and other yoghurts.

## Discussion

This study represents the first complete report on vitamin B_12_ and folate status in Spanish vegetarians. Results reveal sufficient serum vitamin B_12_ levels in the studied subjects independently of their diet option, and the prevalence of clinical vitamin B_12_ deficiency was very low. However, by measuring MMA we detected subclinical deficiency, particularly in the non-users of vitamin B_12_ supplements. Regarding haematological results, the differences between vegans and vegetarians did not reach significance, and macrocytosis was observed in several participants but a clear correspondence with the biochemical biomarkers of vitamin B_12_ was not found.

Elevated MMA was detected in more than 10 % of the population, emphasising the utility of this marker to detect subclinical deficiencies. HHcy was found in more than 30 % of the subjects. The metabolic reactions leading to the production of MMA and Hcy require different cobalamin forms (with methylcobalamin acting in the Hcy pathway and adenosylcobalamin in the formation of succinyl-CoA) and occur in different cell compartments (as the methionine and folate cycles occur in the cytoplasm and the synthesis of succinyl-CoA in the mitochondria)^(^^1^^)^. In this context, previous research has stated that serum vitamin B_12_ might be a misleading marker, as the latency period to detect clinical deficiencies is long^(^^11^^)^. Therefore, we encourage the use of both MMA and Hcy together with serum vitamin B_12_, as each of these biomarkers informs about the action of vitamin B_12_ in different metabolic pathways.

The use of several functional markers is even more important if we consider recent relevant research revealing a relationship between MMA levels and certain genotypes involved in valine catabolism that might affect MMA levels independently of cobalamin status^(^^32^^)^. Therefore the use of Hcy to detect subclinical vitamin B_12_ deficiency through a different pathway is recommended. Consistently, when cobalamin status was assessed considering serum vitamin B_12_, MMA and Hcy simultaneously, the proportion of subjects with values out of range of at least one of the biomarkers was remarkably higher than the proportion detected by using only serum vitamin B_12_. The observed results underline the limitations of serum vitamin B_12_ to detect vitamin B_12_ deficiency and the need of using several functional markers to detect subclinical deficiencies accurately.

Most of the hyperhomocysteinaemic individuals presented mild HHcy (16–30 *µ*mol/l) while only three volunteers had moderate HHcy (31–100 *µ*mol/l)^(^^16^^)^. There is a great variability in the values obtained in European vegetarians; the volunteers in the present study had Hcy levels higher than German vegetarians^(^^15^^,^^33^^)^ and lower than Slovak and Austrian vegetarians^(^^14^^,^^34^^)^. In relation to the type of vegetarian diet, Hcy was higher in lacto-ovo vegetarians than vegans, in contrast to other reports^(^^35^^)^. However, such studies detected lower vitamin B_12_ status in vegans, which has not been observed in our research. We consider that these differences were not detected because of the extended use of cobalamin supplements in both dietary groups.

Looking into other factors to explain Hcy results, Hcy levels are not only influenced by vitamin B_12_, but also by folate status and methionine^(^^14^^)^. Erythrocyte folate was always well above the cut-off value, which is reasonable considering that vegetarian diets are rich in folic acid^(^^2^^)^. Interestingly, erythrocyte folate of more than 50 % of the volunteers surpassed the suggested cut-off for high erythrocyte folate (1360 nmol/l)^(^^36^^)^. This is in agreement with previous research that found higher folic acid intakes in lacto-ovo vegetarians and vegans than in omnivores^(^^33^^,^^37^^)^. Furthermore, the studied subjects consumed fortified products such as vegetable milks that might also contribute to the high folate status. The high levels of folate observed discard folate deficiency as the cause of HHcy^(^^14^^)^. Although the adverse effects of folic acid excess are unknown, an excessive intake of folate might mask cobalamin deficiency symptoms, preventing the increase of Hcy in vegetarian diets and the appearance of haematological symptoms, which may occur in the studied participants^(^^15^^,^^24^^,^^38^^)^.

Regarding the influence of methionine on Hcy, protein consumption and methionine levels have been reported to be higher in lacto-ovo vegetarians than vegans, which in a scenario of folate repletion as observed in our study might lead to higher rates of HHcy in lacto-ovo vegetarian subjects under similar vitamin B_12_ levels^(^^14^^,^^39^^)^. This might explain the higher Hcy levels of lacto-ovo vegetarians compared with vegans detected in this research. It is remarkable that most of the individuals with altered levels of one of the markers only presented Hcy out of range, while MMA was elevated only in eleven individuals. Therefore, we consider that the high intake of methionine with lacto-ovo vegetarian diets might increase circulating Hcy through the methionine cycle, while the formation of MMA and succynyl-CoA would be unaffected. In addition, it should be pointed out that HHcy is a recognised factor of CVD and higher HHcy prevalence has been reported in vegetarians than omnivores^(^^10^^,^^40^^)^, although it seems that vegetarians have lower IHD mortality than non-vegetarians^(^^41^^,^^42^^)^.

In the present study it was clearly shown that vitamin B_12_ supplement users exhibited higher serum vitamin B_12_ and erythrocyte folate, as well as lower MMA and Hcy than non-users; thus the obtained results remark the importance of supplementation both for vegans and lacto-ovo vegetarians. These results are consistent with previous literature considering that the recommended intake of vitamin B_12_ is difficult to achieve by vegetarians if supplements are not consumed^(^^5^^,^^43^^)^, partly because the intake of fortified food items is not enough to provide the required doses of the vitamin^(^^4^^)^.

Considering all the analysed markers, the studied Spanish vegetarians presented sufficient folate and vitamin B_12_ status due to their general supplementation habit. These results contrast with the findings of recent studies on vegetarian Indians, where a prevalence of vitamin B_12_ deficiency of 70 % has been estimated, and more than 50 % of the subjects presented deficient levels of serum vitamin B_12_ and HHcy^(^^44^^,^^45^^)^. This may be explained by the differences in vegetarian diet composition and socio-economic status between these two countries^(^^46^^)^. However, comparing our results with those obtained in other European countries, such as Germany, the Netherlands and UK, the differences remain^(^^15^^,^^33^^,^^43^^)^. We consider that the sufficient vitamin B_12_ status of our subjects might be mainly caused by the extended use of cobalamin supplements in the Spanish participants, while the proportion of vitamin B_12_ consumers found in previous research was smaller. Nevertheless, even the non-users of vitamin B_12_ supplements presented a better status than the lacto-ovo vegetarian and vegan participants of the above-mentioned reports. The better cobalamin status detected in our study might be explained by a higher consumption of vitamin B_12_-rich and vitamin B_12_-fortified food products, and also the age of the volunteers, as vitamin B_12_ status is usually lower in old individuals^(^^1^^)^.

In this regard, the association of vitamin B_12_-rich food with cobalamin status was studied. The results suggest an improvement of serum vitamin B_12_ levels and a tendency to improve Hcy and MMA, associated with the consumption of yoghurts (including yoghurts of animal and vegetable origin, soya yoghurts). Dairy products constitute a source of vitamin B_12_ intake, but they have low concentrations of this vitamin and their positive effects on cobalamin status are due to their elevated consumption^(^^20^^)^. In the studied population, however, it can be appreciated that most of the subjects (eighty-seven volunteers) never consumed animal milk, which could explain the absence of an effect on vitamin B_12_ markers. It is remarkable that thirty-three of these subjects were lacto-ovo vegetarians. Interestingly, it was also observed that twenty-eight of the hyperhomocysteinaemic individuals (thirty-three subjects, thirteen vegetarians) never consumed animal milk, suggesting a relationship between milk consumption and normal Hcy levels. This tendency was also observed for MMA levels, as ten of the eleven deficient individuals belonged to the group of animal milk non-consumers. Regarding the intake of vegetable milks, it was observed that the consumption of these food items was more frequent than that of animal milk. However, vegetable milks did not have any influence on the analysed biomarkers in this research, probably because these milks were not vitamin B_12_-fortified. Furthermore, an association was detected between a higher consumption of eggs and normal Hcy levels. Although the concentration of cobalamin in eggs is high, its absorption is limited, presenting a low bioavailability, which could explain the lack of association with MMA, a specific marker of vitamin B_12_ status^(^^20^^,^^21^^)^. It was observed that sixty-three subjects never consumed eggs, representing most of the population, and only nine of these individuals were lacto-ovo vegetarians.

The categorical character of the collected dietary data and the unbalanced proportion of vitamin B_12_ users and non-users represent limitations of the study. Further research on the topic considering quantitative dietary data must be done to assess the vitamin B_12_ intake through dietary sources, clarify the possible differences in methionine consumption between lacto-ovo vegetarians and vegans, and discover dissimilarities in diet composition with studies developed in other countries. Participants in the present study were not representative of the Spanish vegetarian population. In addition, the influence of polymorphisms in genes involved in the vitamin B_12_ and folate routes was not analysed. However, this research also presents several remarkable strengths, including a sufficient number of volunteers recruited, a balanced distribution of lacto-ovo vegetarians and vegans, and the use of both markers of circulating levels and functional status of cobalamin as previous studies have recommended^(^^11^^)^. Furthermore, the inclusion of haematological markers to detect clinical deficiency, often not considered in studies on cobalamin status, provides a more complete perspective of the issue. Further studies should be focused on the possible effects of high tissue folate and Hcy in this population.

### Conclusions

Vitamin B_12_ status of the studied vegetarian population was generally normal and better than that observed in previous European studies of similar characteristics, while folate status was elevated. The use of both markers of clinical and subclinical deficiency proved to be a useful tool, with 11 % of the subjects presenting subclinical deficiency detected by MMA, and relatively common HHcy. Differences associated with diet option were not detected, observing subclinical deficiencies both in lacto-ovo vegetarians and vegans; thus neither dairy products nor eggs appeared to have a benefit in vitamin B_12_ status. The risk of subclinical deficiency in non-users of supplements was higher, emphasising the need of vitamin B_12_ supplementation in vegetarian diets.
